# Development of a new quantification method using partial volume effect correction for individual energy peaks in ^111^In-pentetreotide SPECT/CT

**DOI:** 10.22038/AOJNMB.2022.61394.1430

**Published:** 2022

**Authors:** Kosuke Yamashita, Noriaki Miyaji, Kazuki Motegi, Takashi Terauchi, Shigeki Ito

**Affiliations:** 1 Department of Nuclear Medicine, Cancer Institute Hospital of Japanese Foundation for Cancer Research, Tokyo, Japan; 2 Graduate School of Health Sciences, Kumamoto University, Kumamoto, Japan; 3 Faculty of Life Sciences, Kumamoto University, Kumamoto, Japan

**Keywords:** Single Photon Emission Computed Tomography (SPECT), ^ 111^In-pentetreotide, Somatostatin receptor scintigraphy (SRS), Indium Uptake Index (IUI) Partial Volume Effect (PVE)

## Abstract

**Objective(s):**

Somatostatin receptor scintigraphy (SRS) using ^111^In-pentetreotide has no established quantification method. The purpose of this study was to develop a new quantitative method to correct the partial volume effect (PVE) for individual energy peaks in ^111^In-pentetreotide single-photon emission computed tomography (SPECT).

**Methods:**

Phantom experiments were performed to construct a new quantitative method. In the phantom experiments, a NEMA IEC body phantom was used. Acquisition was performed using two energy peaks (171 keV and 245 keV) on the SPECT/CT system. The volume of interest was set at each hot sphere and lung insert in the SPECT images of each energy peak, and the recovery coefficient (RC) was calculated to understand the PVE. A new quantitative index, the indium uptake index (IUI), was calculated using the RC to correct the PVE. The quantitative accuracy of the IUI in the hot sphere was confirmed. Case studies were performed to clarify the quantitative accuracy. In a case study, the relationship between the IUI and the Krenning score, which is used as a visual assessment, was evaluated for each lesion.

**Results:**

The obtained RCs showed that the energy peak at 171 keV was faster in recovering the effect of PVE than that at 245 keV. The IUI in the 17-mm-diameter hot sphere was overestimated by 4.8% and 8.3% at 171 keV and 245 keV, respectively, compared to the actual IUIs. The relationship between IUI and Krenning score was rs=0.773 (*p*<0.005) at sum, rs=0.739 (*p*<0.005) at 171 keV, and rs=0.773 (*p*<0.005) at 245 keV.

**Conclusion:**

We have developed a new quantification method for ^111^In-pentetreotide SPECT/CT using RC-based PVE correction for an individual energy peak of 171 keV. The quantitative accuracy of this method was high even for accumulations of less than 20 mm, and it showed a good relationship with the Krenning score; therefore, the clinical usefulness of IUI was demonstrated.

## Introduction

 Neuroendocrine neoplasm (NEN) is a rare disease that occurs in 2.5% of every 100,000 people (1). However, the number of patients with this disease is increasing every year (2). Recently, not only computed tomography (CT) and magnetic resonance imaging (MRI) for morphological diagnosis, but also nuclear medicine examinations using ^111^In-pentetreotide (somatostatin receptor scintigraphy; SRS) is used for the functional diagnosis of NEN (3). ^111^In-pentetreotide binds specifically to subtypes 2, 3, and 5 of the somatostatin receptors (SSTR), which occur frequently and accumulate in NEN, and the accumulation is visualized by a gamma camera (4). Thus, SRS is recommended for use in local diagnosis, metastasis diagnosis, and confirmation of somatostatin receptor expression (5). In addition, peptide receptor radionuclide therapy (PRRT) with a similar mechanism has already been performed by synthesizing the radionuclides ^177^Lu and ^90^Y using a ligand similar to ^111^In-pentetreotide (6). Recently, it has been reported that ^68^Ga-DOTATOC is superior to ^111^In-pentetreotide as a diagnostic method for NEN (7). However, in Japan, the use of ^68^Ga-DOTATOC has not been obtained the pharmaceutical approval and ^111^In-pentetreotide has been widely used in clinical practice. SRS is an important examination to confirm the amount of somatostatin receptors, which affect the efficacy of treatment with PRRT, and can contribute to theranostics expected in the field of nuclear medicine in the future (8). Since no quantitative method has been established for SRS, the Krenning score, which is a visual evaluation, is commonly used (9). ^177^Lu has a physical half-life of 6.7 days by β- decays (0.498 MeV, 78%), emitting γ-rays (208 keV: 11%) (10). 

 In clinical trials using ^177^Lu-DOTA-TATE, the standard of care is to treat patients with a Krenning score of 2 or higher in SRS (11). The Krenning score is a subjective visual score that may not be reproducible (12). Therefore, a quantitative method for ^111^In-SRS is required for clinical studies to improve the quality of diagnosis.

 In an epidemiological study of NENs conducted by Ito et al., a significant correlation (p=0.01) was found between tumor size (>1 cm) and lymph node metastasis, making it clinically important to identify NENs larger than 1 cm (13). However, in SRS, it is difficult to accurately assess the activity of tumors smaller than 2 cm because of the partial volume effect (PVE) caused by the resolution of single-photon emission computed tomography (SPECT) (14). The recovery coefficient (RC) is an index that evaluates the PVE and allows for simple PVE correction (15). Several methods using the recovery coefficient (RC) have been developed to improve the quantification accuracy in SPECT images (16). Additionally, the PVE correction method has been used to improve the accuracy of quantification (17). By applying RC and PVE corrections to the SRS-SPECT, accurate quantitative values can be obtained even for small lesions.

 The energy window setting is also an important factor for SPECT imaging, as it affects the quantitative accuracy (18). ^111^In, which has a physical half-life of 2.8 days, decays by orbital electron capture, emitting γ-rays (171 keV: 90.2%, 245 keV: 94.0%) and characteristic X-rays (Kα: 23.1 keV, 69.0%; Kβ: 26.2 keV, 14.1%) (19, 20). In the case of bone scintigraphy, it has been reported that the quantification accuracy is improved by optimizing the energy window (21). For the SRS-SPECT, the two energy peaks, 171 and 245 keV, are commonly used for imaging (22). Because these peaks have different counting efficiencies of the gamma camera as well as different collimator penetration, scattering correction accuracy, and counting volume, there is a concern that the use of a combined energy window would affect the accuracy of the quantification (23). A more accurate quanti-fication method for SRS can be developed by correcting the PVE by using RC for individual energy peaks. Moreover, this method can be applied to ^177^Lu-SRS.

 Therefore, the purpose of this study was to develop a new quantification method with accuracy by PVE correction, using RC for individual energy peaks calculated by phantom in ^111^In-pentetreotide SPECT.

## Methods

***SRS imaging protocol***

**Figure 1:**
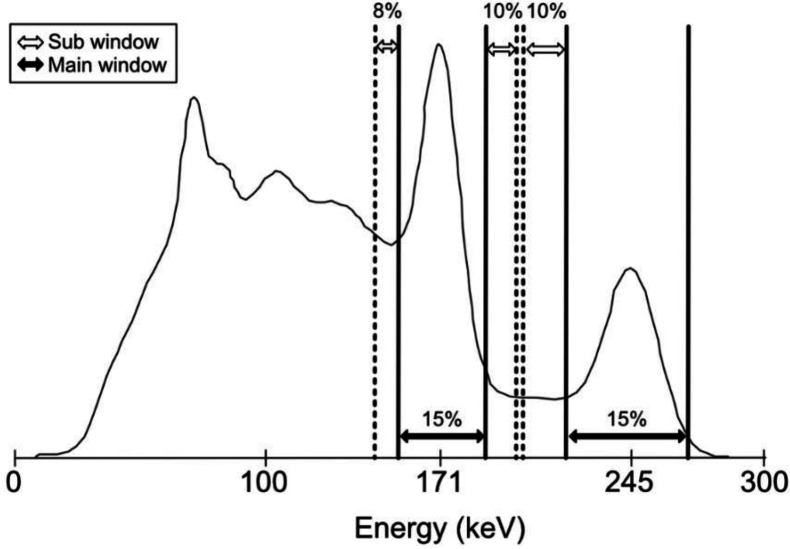
The energy spectrum and window setting of ^111^In

 All images were acquired on a SPECT system (Symbia Intevo 16: Siemens Medical Solution, Erlangen, Germany) with low middle-energy general purpose (LMEGP) collimators. The energy windows for ^111^In-SPECT were 172 keV ±7.5% and 247 keV ±7.5% for the main window. The lower and upper windows at 172 keV were 15% and 8%, respectively, while the lower window at 247 keV was 10% (Figure 1). Projection data for SPECT were acquired for 20 minutes (60 steps, 40 s/step, 128×128 matrix; magnification, 1.0; pixel size, 4.8 mm). SPECT images were obtained by using the ordered subset expectation maximization (OSEM) method (Flash3D; Siemens Healthcare, Erlangen, Germany) with 12 iterations and 6 subsets. Scattering correction was performed using the triple energy window (TEW) method at 171 keV and dual energy window (DEW) method at 245 keV. Attenuation correction was performed using computed tomography attenuation correction (CTAC). The CTAC was performed using an adaptive dose modulation (CARE Dose 4D; Siemens Healthcare, Erlangen, Germany) at a tube voltage of 130 kV and tube current of 80 mA. All images were analyzed using the Daemon Research Image Processor (DRIP; Fujifilm Toyama Chemical Co., Ltd., Tokyo, Japan) version 3.0.2.0, RAVAT (Nihon Medi-Physics Co, Ltd, Tokyo) version 1.00, and OsiriX software (Pixmeo, Bernex, Switzerland) v. 12.0.3.

In this study, the main window (black arrows) of 15% width is opened at the center of 171 keV and 245 keV. The sub-windows (white arrows) of 8% are opened at the lower side and 10% at the upper side of 171 keV, and sub-window of 10% is opened at the lower side of 245 keV

***Phantom studies***

***Phantom design***

**Figure 2:**
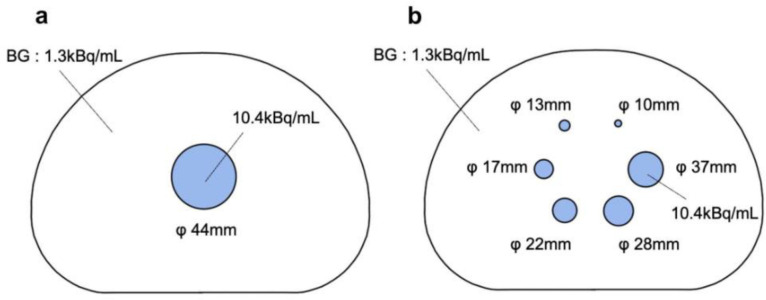
The layouts of the NEMA IEC body phantom

 The characteristics of each energy peak were confirmed by phantom experiments. A NEMA IEC body phantom (Data Spectrum, NC, USA) was used to simulate the upper abdomen. Six hot spheres (φ37, 28, 22, 17, 13, and 10 mm) and lung insert (φ44 mm) were placed in the phantom. The renal excretion rate of ^111^In-pentetreotide in the human body after 24 h is 85%, and the uptake in the upper abdomen relative to the whole body is approximately 60%–70% (22). The activity of ^111^In-pentetreotide in the body after 24 h is approximately 20 MBq. Therefore, the entire phantom contained 13 MBq of the ^111^In solution (Figure 2).

Figure shows the positions of (**a**) lung insert and (**b**) hot spheres (φ37, 28, 22, 17, 13, 10 mm) in the NEMA IEC Body phantom. The lung insert is in the center of the phantom and hot spheres are in concentric circles around the center. The dose of the lung insert and hot spheres was 10.4 kBq/mL and that of BG was 1.3 kBq/mL (a target-to-background ratio (T/B) of 8 (T/B=8/1))

 The cross-calibration factor (CCF), which is necessary to calculate the quantitative value, was measured. A polyethylene bottle (φ95 mm, volume=1170 mL) was used for the CCF; the bottle was filled with 3.47 kBq/mL of ^111^In solution for the measurement. 

***Data analysis***

 Three types of SPECT images were used: images acquired at 172 keV±7.5% and 247 keV±7.5% in the main window (171 keV image and 245 keV image, respectively), and images 

acquired by summing the two windows (sum image). To clarify the PVE, the RC was determined for each window. The IUI was calculated by correcting the RC.

**Figure 3:**
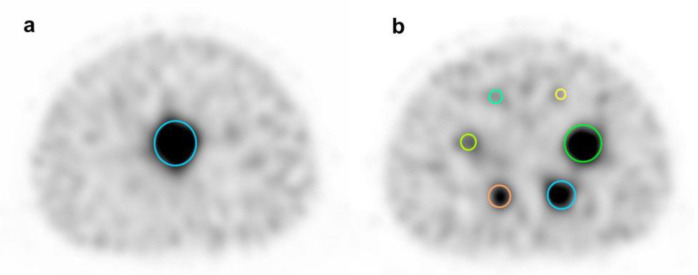
VOI placement in NEMA IEC body phantom

 Volumes of interest (VOIs) were placed at the hot spheres and at the lung insert in the SPECT images for each window (Figure 3). The shapes of the VOIs were spherical for the hot sphere and cylindrical for the lung insert. In the positron emission tomography studies, the peak of standardized uptake value (SUV_peak_) has been shown to be useful in many studies (24, 25).

The VOI was set to the same slice as the hot sphere in the lung insert (**a**); the slice that is most depicted in the hot spheres (**b**)

 Therefore, in this study, the peak value in the accumulation was used to calculate the quantitative value．The maximum value of the mean count of 1 cm^3^ in the VOI for each hot sphere and lung insert was measured and used as the peak count. Jonsson et al. have shown that the RC differs depending on the presence or absence of background activity (26). To reduce the interaction of the background activity, the denominator of the RC calculation in this study was the counts of 44mm accumulations instead of the true activity. The RCs were calculated as follows:

$\mathrm{RC}=\frac{C_{\mathrm{peak},i}}{C_{\mathrm{peak},44}}$ ……… (1)

*C*_peak,44 _is the peak count in the lung insert, and *C*_peak,i_ is the peak count in each hot sphere.

 Based on the relationship between the calculated RCs and the diameters of the hot spheres and lung insert, spline interpolation was performed between the measurements to observe a total of 60 RCs using the original program written in Python. Wilcoxon's signed rank sum test was used to examine the differences between the three measured RCs. The *p*<0.05 was considered statistically significant.

To correct the PVE for accumulations of 44 mm or less in diameter, the peak count of the hot spheres and lung insert must be divided by the RC corresponding to their diameter. In calculating the quantitative value, the dosage and weight of the patient must be considered. 

 The SUV method is commonly used to calculate quantitative values independent of body weight and dose (27). In this study, the IUI was calculated with reference to this method. The IUI of each hot sphere and lung insert was calculated as follows:

$\mathrm{IUI}=\frac{\frac{C_{\mathrm{peak i},\mathrm{energy}}}{{\mathrm{RC}}_{i,\mathrm{energy}}}}{\mathrm{Dose radioactivity}(\mathrm{Bq})/\mathrm{Weight}(g)}\times \mathrm{CCF}$ ……… (2)

*C*_peaki,energy_ is the peak count of each hot sphere in each window, *RC*_i,energy_ is the RC corresponding to the diameter in each window, *Dose radioactivity* is the amount of radioactivity contained in the phantom (Bq), and *Weight* is the weight of the phantom (g). *CCF *is calculated from the radioactivity concentration in the bottle and the counts from the SPECT images.

 In this study, 17 mm spheres were measured to confirm the quantitative accuracy for accumulations of less than 20 mm in diameter. 

 The quantitative values for the 17 mm sphere were calculated using two patterns: IUI and SUV. 

 In addition, the percentage (%) difference was calculated to confirm the difference between the ideal quantitative value and calculated quantitative value. The %difference was calculated as follows:

$\mathrm{\% difference}=\frac{{\mathrm{Index}}_{\mathrm{ref}}-{\mathrm{Index}}_{\mathrm{cal}}}{{\mathrm{Index}}_{\mathrm{ref}}}\times \mathrm{100}$ ……… (3)

*Index*_ref _is the ideal quantitative value of IUI and SUV (IUI, SUV=8.8), and *Index*_ca__l_ is the quantitative value of IUI and SUV by calculation.

***Case study***

***Patient protocol***

 The Ethics Committee at the Cancer Institute Hospital of JFCR approved this clinical study (approval no. 2020-1171). The results of this retrospective study did not influence any further

 therapeutic decision-making.

 All patients underwent SRS at the Cancer Institute Hospital of JFCR between April 2016 and March 2020. Fourteen patients (17 sites) were included in the study. Four patients had normal liver, seven had liver metastases (including two pancreatic tumors), and five had pancreatic, head, and body tumors (including two liver metastases) (Table 1). 

 In all patients, ^111^In-pentetreotide (Octre oscan; Fujifilm Toyama Chemical Co., Ltd., Tokyo, Japan) was administered at an activity of 180.1±14.8 MBq. The selection criteria for patients were as follows: at least one SPECT/CT session performed 24 hours after ^111^In-pentetreotide administration, no liver lesions on modalities other than SPECT/CT in patients with normal liver. In cases of liver metastases and pancreatic head and body tumors, SPECT/CT showed accumulation in the lesions.

**Table 1:** Patient characteristics

**Patient**	**Age**	**Adult**	**Tumor site**	**Lesion size (mm)**	**Krenning score**	**T/B**
1	61	F	Pancreas	35	4	20.62
2	68	F	Pancreas	30	4	64.20
3	52	M	Pancreas	44	3	17.85
4	56	F	Non(Normal Liver)	-	2	-
5	79	M	Non(Normal Liver)	-	2	-
6	36	M	Non(Normal Liver)	-	2	-
7	63	F	Non(Normal Liver)	-	2	-
8	50	M	Liver	25	3	7.41
9	61	F	Liver	13	3	5.53
			Liver	10	3	3.13
			Pancreas	35	4	29.33
10	65	M	Liver	11	3	2.72
			Pancreas	> 44	4	23.45
11	42	M	Liver	14	3	2.20
12	58	F	Liver	13	3	2.18
13	61	F	Liver	> 44	4	15.64
14	83	M	Liver	30	3	3.96

***Data analysis***

 The IUI was calculated for all 17 sites (14 patients) using the RC and cross-calibration factor (CCF) calculated by the phantom experiments. 

**Figure 4:**
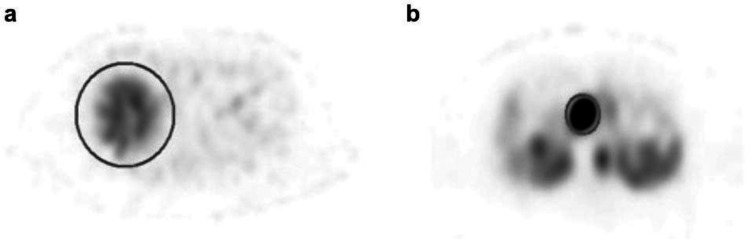
VOI placement in the normal liver and the tumor

 To calculate IUI in normal liver cases, VOIs of a size that enclosed the liver were placed visually in the liver area of the case images (Figure 4a). The IUI in the liver region was calculated for each window using the obtained

 peak count, dose for each case, and body weight.

 For the accumulation of liver metastases and pancreatic tumors, VOIs were set according to the size of each tumor, and the peak counts were measured (Figure 4b). Tumor size was measured using the previous CT and MRI. The RC was selected based on each tumor’s measured length and diameter, and the IUI was calculated using the peak count.

The VOI is placed in a uniform location in the upper part of the liver to enclose the entire liver (**a**). A spherical VOI of the same size as the tumor is placed at the maximum depicted slice of the tumor (**b**)

 Krenning scores were determined for all sites (Table 2). For cases with normal liver, the score was set to 2 based on the criteria of the Krenning score. The correlation coefficient was used to clarify the relationship between the Krenning score and IUI.

**Table 2:** Definition for Krenning score (0-4)

Score	Intensity
0	None (no uptake)
1	Very low
2	Less than or equal to that of the liver
3	Greater than that of the liver
4	Greater than that of the spleen

***Statistical analysis***

To check for differences between the groups, Friedman's test was used for the calculated IUI for each energy window. Box plot analysis was used to clarify the relationship between the Krenning score and IUI, and Spearman's rank correlation coefficient (rs) was calculated. Statistical significance was set at *p*<0.05. All statistical analyses were performed using Easy R (Saitama Medical Center, Jichi Medical University, Saitama, Japan) version 1.54, and the graphical user interface of R (The R Foundation for Statistical Computing, Vienna, Austria) version 3.6.2 (28).

## Results

***Phantom studies***

**Figure 5:**
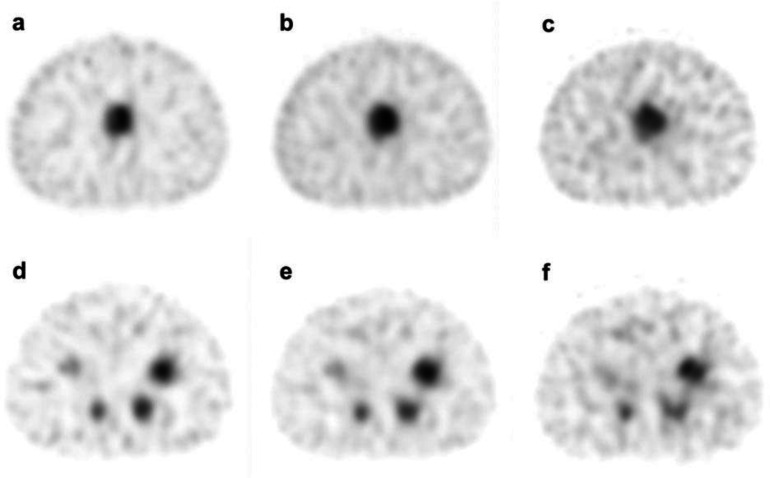
SPECT images of NEMA IEC Body Phantom

 A trans-axial image collected in each window is shown in Figure 5. Compared to the 171 keV image, the 245 keV image shows relatively more noise and a small number of counts around the phantom. In addition, the 171 keV image had the highest image quality, and the accumulation of each hot sphere was circular.

Reconstruction was performed for 171 keV (**a, d**), 245 keV (**b, e**), and sum (**c, f**). The upper image shows the lung insert (**a, b, c**), and the lower image shows the hot spheres (**d, e, f**)

**Figure 6:**
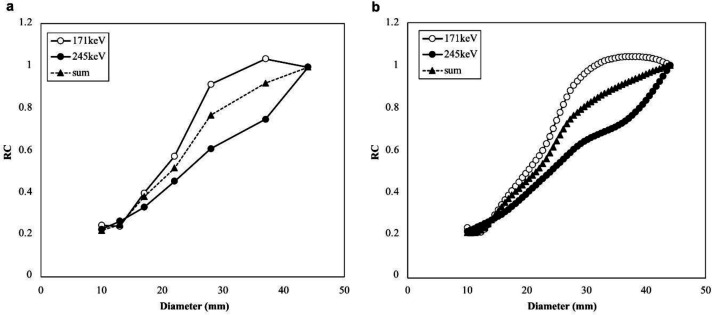
Relationship between the diameter of the accumulation and the RC

 The spline interpolated graphs of the RCs obtained from the NEMA IEC body phantoms for each window are shown in Figure 6. The RCs of the 13 mm and 10 mm spheres were almost the same owing to the PVE. At 171 keV, the RC reached approximately 1.0, at a sphere size of 37 mm, but did not reach 1.0 until 44 mm for the other windows. There was a significant difference between the three RC curves (*p*<0.005).

Table 3 shows the percentage difference in IUI and SUV for the 17-mm-diameter hot sphere in each window. By using RC at the 171 keV or 245 keV energy peaks, respectively, the difference in IUI between the energy peaks was almost eliminated. The SUVs were underestimated by more than 60%. Contrastingly, the IUI at sum, 171 keV, and 245 keV were almost the same as the IUI of 8.8 calculated from the actual radioactivity, but were overestimated by 4.8% at 171 keV and by 8.3% at 245 keV.

(**a**) The RC in each hot sphere and the lung insert. (**b**) The RC with spline interpolation. Triangles indicate sum, white circles indicate 171 keV, and black circles indicate 245 keV data

**Table 3:** Difference (%) between IUI, SUV of each window and ideal IUI, SUV (IUI, SUV=8.8) for lung insert (φ17mm)

Energy Window	IUI	% difference	SUV	% difference
171 keV	8.38	4.8%	3.30	62.5%
245 keV	8.07	8.3%	2.62	70.2%
sum	8.39	4.6%	3.16	64.1%

***Case study***

**Figure 7:**
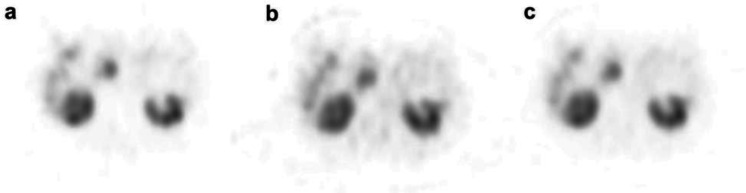
Images of a case with a tumor in the head of the pancreas; (**a**) 171 keV, (**b**) 245 keV, and (**c**) sum

 The median T/B in this study was 7.41 (2.18-64.20). An example of a case image for each window is shown in Figure 7. Similar to the 

results of the phantom experiment, the images using 245 keV showed slight counts along the body surface and bed.

**Figure 8:**
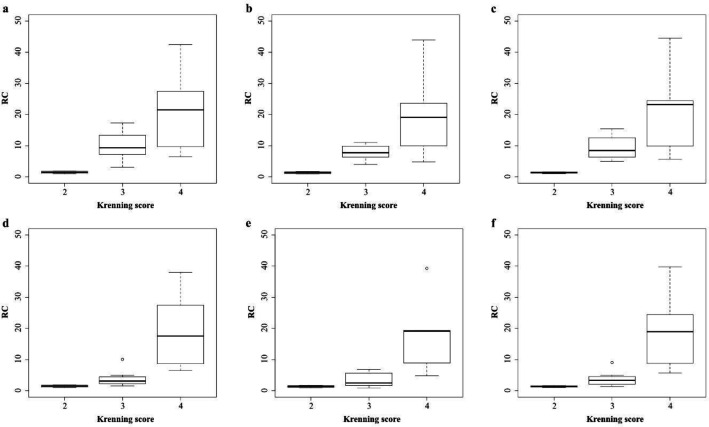
Box plots showing the relationship between Krenning score and IUI (**a, b, c**), SUV (**d, e, f**) in each window; (**a,d**) 171 keV; (**b,e**) 245 keV; and (**c,f**) sum

 The relationship between IUI, SUV, and Krenning score in each window is shown in Figure 8. There was no significant difference in the IUI between the three groups in each window (p=0.056). The relationship between IUI and Krenning score showed strong correlation in each window (sum: rs=0.773, *p*<0.005; 171 keV: rs=0.739, *p*<0.005; 245 keV: rs=0.773, p<0.005). The relationship between SUV and Krenning score also showed strong correlation in each window (sum: rs=0.861, *p*<0.005; 171 kV: rs=0.861, *p*<0.005; 245 keV: rs=0.797, *p*<0.005). The distribution of IUI for each Krenning score was wider than in SUV. 

**Figure 9:**
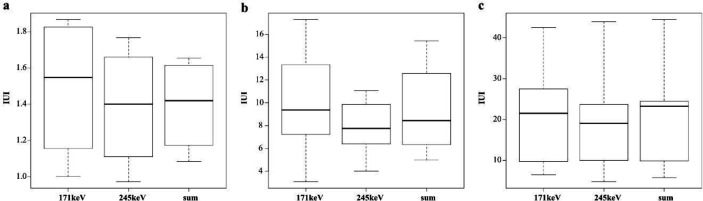
Box plots showing the relationship between IUI and each window in each Krenning score; (**a**) Krenning score 2, (**b**) Krenning score 3, and (**c**) Krenning score 4

 The box plots for each Krenning score are shown in Figure 9. There was no significant difference between the energy windows for each Krenning score (Krenning score 2: *p*=0.105; Krenning score 3: *p*=0.093; Krenning score 4: p=0.819).

## Discussion

 A new quantification method for ^111^In-pentetreotide SPECT was developed using PVE correction with RC values at each energy peak determined by the phantom experiments.

 This study showed that each energy peak of ^111^In has various characteristics. In particular, the shape of the RC curve and the size of the accumulation above 1.0 are different for the two peaks, and they have different characteristics of the PVE. In a previous report, the PVE was not affected when the size of the accumulation was more than twice the system resolution (15). The system resolution of the LMEGP collimator used in this study was 10.4 mm, which means that the PVE was not affected if the accumulation was approximately 21 mm (29). However, the RC did not reach 1.0 until 44 mm in diameter at 245 keV. Holstensson et al. and Noori-Asl et al. confirmed the effect of scattering correction at 171 keV and 245 keV in simulations and phantom experiments and reported that the removal rate of scattered radiation was different between 171 keV and 245 keV (23, 30). In addition, the energy at 5% septal penetration of the LMEGP collimator is 240 keV, and the second energy peak of the ^111^In used (245 keV) was higher (29). 

 The penetration of the energy peak at 245 keV is expected to be more than 5% in the image. 

 In cerebral blood flow using SPECT with ^123^I-IMP, it has been reported that penetration increased the variation in quantitative values (31). 

 Therefore, when the LMEGP collimator was used, the RC was affected by the scattering rejection rate and penetration at the 245 keV energy peak. This result may be a factor that degrades quantification accuracy. Although IUI could provide high quantitative accuracy regardless of the energy peak, the effect of the 245 keV penetration was very pronounced in SPECT images. Mahler et al. reported that penetration using extended low-energy general purpose (ELEGP) was greater than that using collimators for medium energy general purpose (MEGP) (32). Therefore, it is reasonable to use only the 171 keV energy peak for IUI in ^111^In-pentetretoide SPECT to improve the quantitative accuracy and delineation performance.

 Currently, there are many reports on the use of SUV in bone SPECT examinations (33). SUV has the problem of PVE, which is limited by the inherent system resolution of the device, and it is difficult to calculate accurate quantitative values (34). Tran-Gia et al. performed PVE correction using RC in ^177^Lu-SPECT and reported its usefulness (16). Applying the same method to SRS-SPECT, the activity quantification simply reduces the PVE inherent to gamma cameras. It has been reported that the quantitative accuracy of bone SPECT is within 3% by improving the accuracy of various corrections (35). The activity of SRS is lower than that of bone SPECT. This difference increases the noise, which may result in lower quantitative accuracy. However, in a phantom experiment simulating the actual dose, the IUI established a quantitative accuracy of approximately 5%, suggesting the usefulness of PVE correction using RC. 

 The distance between the subject and the detector is closely related to the resolution of SPECT images (36). Tokorodani et al., in a phantom experiment, reported different SUVs depending on the distance between the subject and the detector (37). Additionally, we previously reported that the RC values differed depending on the positioning of the patient in 

bone SPECT (38). Both the phantom and patients SPECT images were obtained by a non-circular orbit projection in this study. Therefore, the same orbit distance in the phantom experiment is required for the clinical study to obtain stable quantitative values.

 A good relationship was observed between IUI and Krenning score, as shown in Figure 8. This indicates the clinical usefulness of IUI. The Krenning score has been used in clinical trials of therapeutic agents such as PRRT and lanreotide (6, 39). The Krenning score has also been reported to be a strong indicator of the therapeutic effect of PRRT (40). Therefore, it may be difficult for the IUI to replace the Krenning score. In this study, the IUI showed a wide range of values for each Krenning score and was able to subdivide the characteristics of the lesions. Therefore, IUI should be used as a complement to the Krenning score.

 These results suggest that IUI is a clinically useful tool for improving the quality of diagnosis using ^111^In-pentetreotide SPECT. In addition, the fact that the IUI can be calculated simply and without the need for special devices is one of the greatest advantages of this quantitative method, and we believe that it is feasible to verify the proposed quantitative method in a large-scale multicenter study.

 In this study, the ME collimator used in ^111^In-pentetreotide SPECT was not examined. In ^111^In-pentetreotide SPECT, the difference in delineation performance between ME general purpose (MEGP), ELEGP, and low energy general purpose (LEGP) has been reported (32). The characteristics of the energy peaks are expected to differ from those of LMEGP. The use of IUI in the case of the ME collimator is a matter for further study.

 The RC value fluctuates depending on the effect of spill-in from background to the tumor (41). Sakaguchi et al. proposed a PVE correction using the RC considering the T/B and tumor diameter (42). This study calculated the RC in ^111^In-pentetreotide SPECT by setting the T/B value at 8. Furthermore, the peak value (the maximum value of the mean counts when searching 1-cm^3^ sphere in the volume of interest [VOI]) was adopted instead of the maximum value to reduce the influence of the spill-in. The median T/B was 7.41, indicating the middle of the distribution. Thus, a T/B of 8, which was used experimentally, was a reasonable basis for setting the T/B. However, the influence of the spill-in was not eliminated in this study. Further study is necessary to clarify the influence of spill-in.

 All IUIs were calculated using a manual VOI setting, and where the VOI setting position differs each time, the reproducibility of quantitative values could not be maintained. Therefore, it is expected that the development of an automatic VOI setting program will improve the reproducibility and the analysis efficiency of the proposed IUI calculation; thus, further studies are required in this regard.

## Conclusion

 We developed a new quantification method for ^111^In-pentetreotide SPECT. This method has high quantification accuracy even for accumulations of less than 20 mm by PVE correction using RC for an individual energy peak of 171 keV. In addition, the IUI has a good correlation with the Krenning score, which has been used in the past, and is expected to be used as a complementary method to the Krenning score in the future.

## Abbreviations

PVE: Partial Volume Effect

SPECT: Single Photon Emission Computed Tomography

RC: Recovery Coefficient

IUI: Indium Uptake Index 

NEN: Neuro Endocrine Neoplasm

SRS: Somatostatin Receptor Scintigraphy

SSTR: Somatostatin Receptor

PRRT: Peptide Receptor Radionuclide Therapy 

LMEGP: Low Middle Energy General Purpose

OSEM: Ordered Subsets Expectation Maximization

TEW: Triple Energy Window

DEW: Dual Energy Window

CTAC: Computed Tomography Attenuation Correction

DRIP: Daemon Research Image Processor

VOI: Volume of Interest

CCF: Cross-Calibration Factor

SUV: Standardized Uptake Value

TB: Target-to-Background

ELEGP: Extended Low Energy General Purpose

MEGP: Medium Energy General Purpose

LEGP: Low Energy General Purpose

## Ethical considerations

 The Ethics Committee at the Cancer Institute Hospital of JFCR approved this clinical study (approval no. 2020-1171). The results of this retrospective study did not influence any further therapeutic decision-making.

## Availability of data and material

 All data generated or analyzed during this study are included in this published article.

## Competing interests

 The authors declare that they have no competing interests.
